# Pain Management and Its Possible Implementation Research in North Ethiopia: A before and after Study

**DOI:** 10.1155/2020/5317352

**Published:** 2020-06-02

**Authors:** Mengistu Hagazi Tequare, James John Huntzicker, Hagos Gidey Mhretu, Yibrah Berhe Zelelew, Hiluf Ebuy Abraha, Mehari Abrha Tsegay, Kesatea Gebrewahd Gebretensaye, Daniel Gebre Tesfay, Julio Gonzalez Sotomayor, Rahel Nardos, Mary Beth Yosses, Joshua Edwin Cobbs, Jennifer Pui Ling Schmidt, Wendy Weisman, Leslie K. Breitner

**Affiliations:** ^1^Mekelle University, College of Health Sciences, School of Public Health, Mek'ele, Ethiopia; ^2^Oregon Health and Science University, Portland, OR, USA; ^3^Mekelle University, College of Health Sciences, School of Medicine, Mek'ele, Ethiopia; ^4^Mekelle University, Ayder Comprehensive Specialized Hospital, Mek'ele, Ethiopia; ^5^McGill University, Desautels Faculty of Management, Montreal, Canada

## Abstract

**Background:**

Though there is an effective intervention, pain after surgical intervention is undermanaged worldwide. A systematic implementation is required to increase the utilization of available evidence-based intervention to manage the inevitable pain after surgery. The aim of this research project is to develop a scalable model for managing pain after cesarean section by implementing the World Health Organization's (WHO) pain management guidelines through a combination of implementation research and quality improvement methods.

**Methods:**

We implemented the World Health Organization (WHO) pain management guidelines using effective implementation strategies. First, we conducted a formative qualitative exploration to identify enablers and obstacles. In addition, we took base-line assessment on pain management implementation process and outcome using a checklist prepared from the guideline and an adapted American Pain Outcome assessment tool version 2010, respectively. Then, we integrated the guidelines into the existing practice by using collaborative iterative learning strategy. We analyzed the data by Statistical Packages for Social Sciences (SPSS) version 21. We compared the before and after data using chi-squared and Fischer's exact test. A change in any measurement was considered as significant at *p* value 0.05.

**Result:**

We collected data from 106 mothers before and 110 mothers after intervention implementation. We successfully integrated pain as a fifth vital sign in more than 87% (*p* value <0.001) of patient, and fidelity was approximately 59% (*p* value <0.001). In addition, we significantly improved pain outcome measures after the implementation of the intervention. *Conclusion and Recommendations*. A systematic approach to implement pain management guidelines was successful. We recommend the ward sustain these gains and that hospital, the region, and the nation to replicate the success.

## 1. Introduction

There are continuing advancements in multimodal analgesia techniques to prevent and control pain after surgical interventions [1–6]. Though solutions are available, patients undergoing surgery still suffer from postoperative pain of varying degrees in low- and high-income countries across the globe [6–12]. A study conducted in the United States reported that 80% of patients who underwent surgery experienced pain after surgery, of which 86% had moderate, severe, and extreme pain [7]. Another study from China revealed that around 85% patients complained of pain after surgery [8]. In addition, a study conducted in one Ethiopian Tertiary care hospital found more than 91% patients who had a surgical intervention experienced pain [11]. A similar study in another tertiary care hospital of Ethiopia also showed 78% of postoperative patients suffering from pain ranging from moderate to severe intensity [12].

Poorly managed postoperative pain may result in the development of chronic postoperative pain, impaired function, and delayed recovery from surgery. In addition, it could cause depression, mood alterations, sleep disorders, inability to focus, abnormal appetite, poor hygiene, and prolonged opioid use. As a result, poor postoperative pain management increases the medical costs to the patient and the health system [13–16].

Pain after surgery is mostly predictable [17]. Different factors could predict pain of post operation: preoperative state anxiety, level of preoperative pain, preoperative information, age, gender, type of surgery, incision size, ethnicity, and education [18–21].

There are barriers that hinder utilization of the well-known practice. Attitude and aptitude (knowledge and skill) of practicing professionals, low leadership focus, drug regulatory and legislative rules, inadequate resource-like staffing, equipment, and financial constraints in low-income nations are among the barriers for implementation [22, 23].

Evidence shows different approaches at different hierarchies to overcome the barriers of pain management. Making pain management a priority, modifying policies for drug supply management and prescription pattern, collaboration among multidisciplinary professionals, job training for the health practitioners, improvement of pain management education in under and postgraduate curriculum, identifying a working list of drugs for pain and quality improvement approaches are among well-recognized strategies [13, 22–30].

The aim of this research project is to develop a scalable model for managing pain after caesarean section by implementing the World Health Organization's (WHO) pain management guidelines through a combination of implementation research and quality improvement methods.

## 2. Materials and Methods

### 2.1. Design

We employed a before and after intervention study design to implement effective pain management protocol. In addition, we utilized iterative collaborative quality improvement methods to integrate the new protocol with the existing system.

### 2.2. Study Area and Period

The study was conducted in the Ayder Comprehensive Specialized Hospital labor ward, north Ethiopia, from January to July 2018. The ward has 18 postoperative beds, 11 obstetrician/gynecologists, 50 midwives, and 7 interns. The total number of caesarean sections conducted in 2017/2018 (one Ethiopian Fiscal year) was 1490. Within the study period, there were a total of 800 caesarean section procedures conducted [31].

### 2.3. Sample Size

We took samples of mothers who underwent caesarean Section 3 weeks before and 3 weeks after the implementation. We employed consecutive sampling technique. Patients who were unwilling to participate, unconscious during data collection, unable to clearly communicate ideas, and known psychiatric illness were excluded. Before the intervention, we found 106 mothers underwent caesarean section and 110 mothers after intervention.

### 2.4. Procedures

We collected four categories of variables: sociodemographic, clinical, pain management process, and pain outcome variables. For the first three, we prepared a piloted data extraction sheet and collected the data from patients' medical record. For the outcome variables, we adapted the American Pain Society Pain Outcome Questionnaire (APS-POQ-R) version 2010 [32] contextually by piloting it in 5 patients, which is around 5% of the prestudy sample size. The reason why we are using this tool is because many studies used it as an outcome pain measurement tool [11, 33]. The APS-POQ-R was translated into local languages (Tigrigna and Amharic) before starting data collection. Furthermore, we changed the 11-level pain scoring scale into four-level scoring scale (no pain, mild pain, moderate pain, and severe pain) after testing in five patients for easy understanding. The pain outcome data were collected by interviewing patients approximately 24 hours after they underwent surgery.

### 2.5. Intervention Core Components

**Figure 1:**
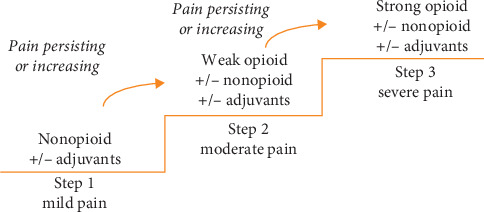
A pocket guide of pain management in Africa (source: beating pain) [22].

The intervention is the WHO pain management guidelines. The implementation of effective pharmacologic pain management will involve the inclusion of pain assessment as a fifth vital sign and provision of pharmacologic solutions (Figure 1).

The core components are as follows:

- Inclusion of pain as a fifth vital sign—the frequency of pain recorded as a fifth vital sign should be equal to the frequency of the four other vital signs
- Drug treatment based on the protocolas shown in Figure 1

### 2.6. Implementation Strategies

We used the following strategies to implement the intervention based on recommendations from the literature [34] and our qualitative formative research:

1. Develop stakeholder interrelationships
2. Train and educate stakeholders
3. Change infrastructure
4. Use evaluative and iterative strategies

### 2.7. Develop Stakeholder Interrelationships (Teams)

To effectively implement our strategy, we formed two teams: research team and quality improvement team. Both teams performed their roles independently without the knowledge what the other team was doing. The principal investigator coordinated the overall project for the project so that the project would go smoothly and that the activities in the proper sequence.

#### 2.7.1. Role of Research Team

The role of the research team was to collect baseline and end-line data, enter, clean, analyze, and report. This team was led by the principal investigator.

#### 2.7.2. Role of the Quality Improvement Team: Iterative Strategy

The quality improvement team prepared the postcaesarean section pain management protocol; prepared necessary resources, like drugs and vital sign sheets; and provided training and orientation for all midwives, medical residents, and interns about the protocol before implementation. Furthermore, the team trained the newly incoming residents, interns, and midwives during implementation.

The other role of this team was collecting data daily from all postoperative mothers on three core measures: inclusion of pain as a fifth vital sign, correct drug treatment, and mothers' satisfaction on pain management. The principal investigator followed the process closely daily and provided necessary feedback and learned from actual practice. We used model for improvement [35] for iterative learning. In addition, learning from each change ideas developed to integrate pain management into the existing system, and sketching the information collected in run charts for decision-making [36] was one of the major roles of the team. The main change ideas developed were training staff on the protocol and revising vital sign sheet to include pain.

### 2.8. Train and Educate Stakeholders

After preparation of the pain management protocol, members of the team trained the midwives, interns, and residents. These are frontline workers who are directly involved in the patient care.

### 2.9. Change Infrastructure

We added pain as a vital sign in the vital sign sheet and pain in the training/orientation list for midwives and medical students. In addition, we added pain management in the evaluation criteria for students and midwives.

In summary, through the full participation of the staff working in the area, we included pain as a fifth vital sign using iterative multidisciplinary learning.

### 2.10. Data Analysis

The data were entered into Microsoft Excel version 2010. Then, it was transferred into Statistical Packages for Social Sciences version 21 for analysis. We cleaned, coded, and checked for accuracy before analysis. Following this, we compared sociodemographic and clinical characteristics of patients and process and outcome variables of the pain management before and after our intervention. We used chi-squared tests for those which fulfil the assumptions and Fischer's exact test for cells containing less than 5 expected values. *p* value less than or equals to 0.05 was taken as a statistically significant change.

## 3. Results

### 3.1. Implementation Process and Strategies

We used four implementation strategies in our implementation process:

1. Develop stakeholder interrelationships: we formed two teams, research team and quality improvement team.
2. Train and educate stakeholders: we trained 50 midwives, 24 interns, and 7 residents
3. Change infrastructure: we changed vital sign sheet, training list, and evaluation criteria
4. Use evaluative and iterative strategies

We used model for improvement for continuous pain management process and outcome improvement. We took baseline data for three key performance indicators (proportion of patients' pain included as a fifth vital sign, proportion of patients correctly treated, and proportion of postcaesarean section mothers satisfied by pain management) for 18 days. Then, we started implementing the protocol, collected data daily, gave feedback, and followed this for 37 days. A total of 683 patient interactions were made to collect data for 55 data points for the whole improvement process.

### 3.2. Pain Management Process and Outcome Improvement

#### 3.2.1. Pain Management Process Improvement

We implemented pain management protocol for postcaesarean section mothers for six months, and we approached 106 and 110 mothers before and after the intervention, respectively. The participation rate in both situations was 100%. The pain management process was significantly improved: the inclusion of pain as a fifth vital sign was improved from none to 87.30% and correct pharmacologic treatment (fidelity) of pain as the protocol was improved from 25.5% to 59.1% (Table 1).

#### 3.2.2. Pain Management Outcome Improvement

Pain outcome was significantly improved after implementation of the pain management protocol in all parameters when compared with the condition before intervention (Table 2). In a four-level Likert scale, the least pain patients experienced within 24 hours after surgery; patients reported “moderate” pain reduced from 20.8% to 0.9%. In addition, patients experienced worst pain reported “severe” reduced from 52.8% to 17.3% and the amount of time patients spent in severe pain reported as “always” and “around half” reduced from 16.0% to 0.9% and from 61.3% to 34.5%, respectively.

Restriction of activities and interruption of normal life due to pain was also significantly improved. Proportion of patients reporting severe and moderate restriction in bed reduced from 23.6% to 3.6% and from 60.4% to 38.2%, respectively. In addition, proportion of patients reporting severe restriction when moving out of bed reduced 49.1% to 18.2%. Moreover, proportion of patients reporting moderate interference in falling asleep reduced from 45.3% to 3.6% and interference in staying asleep reduced from 30.2% to 1.8%.

The effect of pain on mood and emotions of patients was also significantly improved. Proportion of patients reporting moderate anxiety due to pain reduced from 72.6% to 9.1%. Patients reported mild and no for depression, frightening and helplessness before and after the intervention. No patient reported moderate or severe in both times. But, three of them were improved significantly from mild to no.

Drug side effects showed slight change before and after the implementation. From four parameters, only drowsiness showed significant improvement. Proportion of patients reported moderate drowsiness reduced from 2.8% to 0.9%. Nausea/vomiting, itching, and dizziness showed no significant change.

Pain relief and patients' satisfaction showed significant improvement after the intervention. Proportion of patients whose pain relieved completely before and after the intervention improved from 3.8% to 23.6% and most relief improved from 26.4% to 60.0%. In addition, proportion of patients participated in their pain treatment as much as they wanted increased 30.2% to 80.9%. Finally, the proportion of patients who were satisfied by the pain management made to them improved from 34.0% to 80.9%.

## 4. Discussion

### 4.1. Implementation Strategies

We used proven implementation strategies [37] to integrate pain management protocol in the daily routine practice in ACSH labor ward. The strategies are as follows: building change coalition, training the staff with the intervention components (including the coalition members), changing some recording and performance management systems, and using iterative quality improvement methods.

We started the improvement process by forming two teams. Those teams were informed about the criticality of the pain management problem and the aim of the overall project. This is in line with Kotter's model for managing change [38]. Kotter describes eight consecutive phases to happen to bring about a change. The first and second are creating a sense of urgency and bringing people together to have a shared understanding, respectively.

In implementing a new intervention, the first process that should happen is creating awareness about the intervention. According to Roger's diffusion of innovation, the decision to adopt a new thing starts by knowing its existence and its function and the innovation decision process continues based on the effect of the knowledge [39]. Based on this principle, the implementation team in this study conducted training for the staff on the pain management protocol. The training was done by themselves to increase ownership.

The training was followed by implementation. The implementation team had to change some infrastructure, like recording [37]. We inserted the change into the existing record sheet to avoid additional burden to the staff. Every change was done by the full participation and ownership of the implementing team. Real change not only happens in the real work but also in those who do the job and do the change and people own what they create [40]. This helps innovations to be easily adopted and sustained. Furthermore, we used the principles of Edgar Schein about process consulting. Schein advises to help people help themselves and help them release their potential to understand their own problems and solve their problems themselves, not prescribing solutions [41]. Henry Mintzberg strengthens this by arguing that a strategy in health care should come/comes from the frontline staff [42].

The iterative quality improvement strategy helped us repeatedly test and learn and finally integrate our intervention to the existing system of care. We tested it in small scale and learned the constraints on a focused manner. We could learn more in small scale and were able to predict to full scale later. These types of strategies are effective ways for adaptations of evidence-based medicine nowadays [23, 37].

After and during the implementation of all these strategies, we could see a significant change in the process and outcome metrics of pain management after caesarean section. This may be due to our intervention.

### 4.2. Pain Management Process and Outcome

There was a significant improvement of pain management process and outcome after the implementation of this study. The improvement could be attributed to this study. Pain as a vital sign was included in majority of the patients (Table 1), but it was not 100%. This may be due to a difference in patient characteristics. Some patients had a different vital sign sheet, and this might have contributed to this gap. Even though there was a significant improvement in the correct treatment of pain based on the WHO ladder (the protocol) [22], there remains a huge gap between the recommended treatment and the practice.

Pain management outcome, as measured from the patients' reports, has significantly been improved. Pain experience within 24 hours after surgery was improved after the study (Table 2). Majority of the patients experiencing the least pain after the study shifted to mild and no pain. In addition, the worst pain experienced within 24 hours after surgery also changed from severe to moderate and mild pain. Our result is far better than other studies conducted in China and Ethiopia [11, 43], showing most patient experiencing moderate to severe pain after surgery. This difference could be due to our intervention. In fact, before our pain improvement, 96.2% of patients experienced moderate and severe pain, which is similar to the result of the abovementioned study in Ethiopia. The pain significantly decreased after intervention in our study (85.5%)

Mood or emotional alterations due to severity of pain (anxiety, depression, frightening, and helplessness) have been significantly improved after our intervention. Majority of the patients were experiencing moderate anxiety before our study and more than 90% felt mild or no anxiety after our intervention.

The interruption of function or limitation of activities (movement in the bed, moving out of bed, falling asleep, and staying asleep) due to pain of patients after surgery has significantly been improved after our intervention.

However, there was no significant difference in side effects (nausea/vomiting, dizziness, itching, and drowsiness) of pain drugs except for drowsiness, which showed a significant change. The result is acceptable. Because we did not do anything to affect drug side effects and also, we did not add a new drug into the care. The only significant change seen is drowsiness, and this may be due to opioid introduction of the regimen.

The amount of pain relief with the pain care improved significantly. Furthermore, participation of patients on their own care is one of the parameters of patient-centered care [44]. In our study, we tried to improve the participation of patients in their own pain management by active inquisition. Finally, the study improved patients' participation the pain management significantly. The overall satisfaction of patients in the pain management process changed significantly, showing strong possible relationship with participation in implementation of the new pain management protocol.

## 5. Conclusion

We implemented an effective pharmacologic pain management protocol in an Ethiopian Tertiary Care Hospital labor ward for postcaesarean section patients successfully. Taking pain as a fifth vital sign was successfully integrated into the existing system. Consequently, pain outcome experienced by postcaesarean section patients after implementation of pain management protocol was significantly improved when compared with the baseline information.

## Figures and Tables

**Table 1:** Process variables of postcaesarean section mothers before and after implementation of pain management protocol, ACSH, north Ethiopia, Jan–June 2108.

Characteristics	Before intervention		After intervention		*p* value
	*N* (106)	%	*N* (110)	%	
Pain as fifth vital sign					
Yes	0	0.0	96	87.3	<0.001
No	106	100.0	14	12.7	

Correct treatment					
Yes	27	25.5	65	59.1	<0.001
No	79	74.5	45	39.9	

**Table 2:** Outcome variables of pain management implementation for postcaesarean section patients in ACSH, north Ethiopia, Jan–June 2018.

Characteristics	Before intervention		After intervention		*p* value
	*N* (106)	%	*N* (110)	%	
Least pain experienced within 24 hours					
Moderate	22	20.8	1	0.9	<0.001
Mild	80	75.5	91	82.7	
No	4	3.8	18	16.4	

Worst pain experienced within 24 hours					
Severe	56	52.8	19	17.3	<0.001
Moderate	46	43.4	75	68.2	
Mild	4	3.8	16	14.5	

Amount of time spent in severe pain within 24 hours					
Always	17	16.0	1	0.9	<0.001
Around half	65	61.3	38	34.5	
Some of it	24	22.6	71	64.5	

Restricting activities in bed					
Severe	25	23.6	4	3.6	<0.001
Moderate	64	60.4	42	38.2	
Mild	17	16.0	64	58.2	

Restricting activities out of bed					
Severe	52	49.1	20	18.2	<0.001
Moderate	44	41.5	75	68.2	
Mild	10	9.4	15	13.6	

Interferes falling asleep					
Moderate	48	45.3	4	3.6	<0.001
Mild	52	49.1	97	88.2	
No	6	5.7	9	8.2	

Interferes staying asleep					
Moderate	32	30.2	2	1.8	<0.001
Mild	60	56.6	91	82.7	
No	14	13.2	17	15.5	

How much the pain makes you feel anxious					
Moderate	77	72.6	10	9.1	<0.001
Mild	23	21.7	78	70.9	
No pain	6	5.7	22	20.0	

How much the pain feel you depressed					
Mild	78	73.6	30	27.3	<0.001
No	28	26.4	80	72.7	

How much the pain makes you feel frightened					
Mild	46	43.4	2	1.8	<0.001
No	60	56.6	108	98.2	

How much the pain makes you feel helpless					
Mild	21	19.8	0	0.0	<0.001^*∗*^
No	85	80.2	110	100	

Severity of nausea/vomiting					
Mild	42	39.6	36	32.7	0.323
No	64	60.4	74	67.3	

Drowsiness					
Moderate	3	2.8	1	0.9	0.01
Mild	27	25.5	12	10.9	
No	76	71.7	97	88.2	

Itching					
Moderate	56	52.8	10	9.1	0.543
Mild	23	21.7	78	70.9	
No pain	6	5.7	22	20.0	

Dizziness					
Mild	15	14.2	18	16.4	0.863
No	91	85.8	92	83.6	

How much pain relief was receives in 24 hours					
All	4	3.8	26	23.6	<0.001
Around half	28	26.4	66	60.0	
Some	74	69.8	18	16.4	

Were you allowed to participate in pain treatment as much as you wanted?					
Yes	32	30.2	89	80.9	<0.001
No	74	69.8	21	19.1	

Are you satisfied with the result of your pain treatment?					
Yes	36	34.0	89	80.9	<0.001
No	70	66.0	21	19.1	

^*∗*^ = Fischer's exact test used.

## Data Availability

Data used to support the findings of this study are available from the corresponding author upon reasonable request.

## Abbreviations

APS-POQ-R: American Pain Society Pain Outcome Questionnaire
ACSH: Ayder Comprehensive Specialized Hospital
WHO: World Health Organization.
